# Post-harvest biology and recent advances of storage technologies in sugarcane

**DOI:** 10.1016/j.btre.2022.e00705

**Published:** 2022-01-30

**Authors:** Varucha Misra, AK Mall, S Solomon, Mohammad Israil Ansari

**Affiliations:** a ICAR-Indian Institute of Sugarcane Research, Lucknow, U.P., 226002 India; b Chandra Shekhar Azad University of Agriculture and Technology, Kanpur U.P., 208002, India; c Department of Botany, University of Lucknow, Lucknow, U.P., 226007 India

**Keywords:** Secondary losses, Microbes, Sucrose, Cane weight, Management

## Abstract

Sugarcane deteriorates at a quick rate, just like other perishable crops. The quick loss of sucrose content in sugarcane from the time it is harvested has a significant impact on sugar recovery. This problem of post-harvest sucrose losses in sugarcane is a serious concern in cane-producing countries, as it not only leads to low sugar recovery in mills, but also to poor sugar refining. Unreasonable delays in cane transportation from the fields to the mill are frequently linked to a number of problems related to primary or secondary sucrose losses, all of which contribute to a significant reduction in cane weight and sugar recovery. In sugar mills, the processing of damaged or stale canes also presents a number of challenges, including increased viscosity due to dextran generation, formation of acetic acid, and dextrans due to *Leuconostoc* spp. invasion, and so on. The combination of all of these variables results in low sugar quality, resulting in significant losses for sugar mills. The primary and secondary losses caused by post-harvest sucrose degradation in sugarcane are enlisted. The employment of physico-chemical technologies in farmers' fields and sugar mills to control and minimize these losses has also been demonstrated.

## Highlights

• Post-harvest sucrose losses are a vital issue in sugarcane for farmers and sugar millers.
• Microorganisms are an important player in post-harvest sucrose losses in sugarcane.
• Time duration management of cut to crush is one of the controlling factors for postharvest quality in sugarcane.
• Biocidal, chemical, eco-friendly compounds, and chemical formulation assist in minimizing the post-harvest quality losses.

## 1 Introduction

**Fig. 1:**
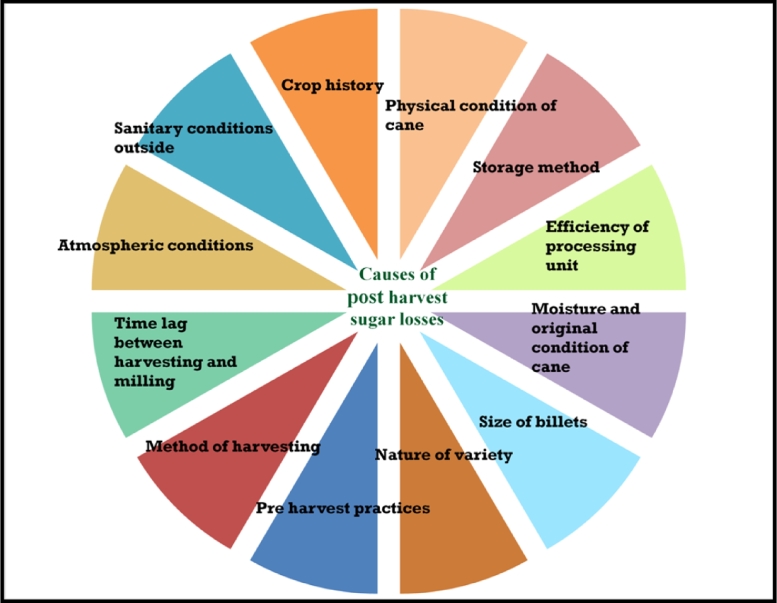
Twelve causes responsible for post-harvest sucrose losses and low sugar recovery.

Post-harvest sucrose losses are an imperative problem in sugarcane for farmers and sugar millers alike. A significant percentage of sucrose is lost when farmers leave harvested canes in fields for several days (to prepare the field for the following crop) or during transportation to mills. The degradation of sucrose in canes begins as soon as the cane is harvested. These losses become more pronounced as the time between harvesting and milling increases. According to Wood et al. [145], a three- to four-days delay in harvesting to crushing is a regular occurrence in the sugar industry. As the quality of the cane deteriorates, the sugar recovery decreases, resulting in economical losses to sugar industries. Sugarcane post-harvest sucrose losses can be high or low depending on a variety of circumstances (Fig. 1). Ambient temperature, humidity, soluble invertase activity, cane variety, and maturity status are all factors that millers frequently overlook during the management process [119, 120, 122]. Furthermore, the formation of undesired chemicals during cane processing (due to chemical or microbiological activity) adds to these losses and causes challenges in sugar processing, culminating in uneconomical sugar manufacturing [95, 121].

The reduction in sucrose content over the time following cane harvest results in low sugar recovery, reducing mills' economies, making post-harvest losses a topic to consider and comprehend. The primary and secondary losses that cause post-harvest sucrose losses have been briefly described here. The numerous management methods that must be used to manage these losses have also been detailed.

## 2 Economical losses

Sugar mills' economies suffer greatly when cane processing is delayed or stale cane is processed (Fig. 1). According to Solomon [117], canes supplied to Indian sugar mills are mostly deteriorated with about 1/4th of the canes obtained being of stale quality, resulting in a loss of approximately Rs. 1600 crores to sugar mills. According to Perry et al. [99], this problem costs the South African sugar industry $6 million per year. A hypothetical sugar mill situation found that crushing the stale canes (storage of more than 72 h) might result in a loss of Rs. 3 to 5 lakh per day for a sugar mill with a capacity of 5000 TCD (Solomon, 2009). According to Rao [101], there is a loss of 2% in sugar recovery when the cane is stored for more than 72 h, and a loss of 7.7 kg/tcg in sugar recovery when cane processing is delayed for 1 h [15]. Furthermore, Solomon [121] demonstrated that there is a loss of one unit in terms of pol percent from harvesting to milling. Additionally, the range of quality losses caused by crushing stale canes has been discovered to be between 12 and 50 percent (in sugar recovery). When one ton i.e., 1000 kg of cane is crushed according to the typical amount of crushing in Indian sugar mills, an approximate loss of 5 to 10 kg of sugar has been documented. This is enhanced when mill crushing periods are extended due to adverse conditions (especially high temperatures during summer seasons).

## 3 Factors affecting sucrose losses after harvest and low sugar recovery

Post-harvest sucrose losses are governed by two factors, both of which result in limited sugar recovery.

1. Primary losses: Primary losses occur when sucrose inversion happens spontaneously after cane harvest or when the cane is left over standing for an extended period of time. The primary losses are compounded by delays in cane delivery owing to poor transportation. Varietal differences influence primary losses as well.

2. Secondary losses: Secondary losses are losses that occur indirectly, such as those produced by the development of dextran, alcohol, acids, and other substances in juice recovered from cane as a result of inefficient and unhygienic cane processing. Chemical (acid), enzymatic, and microbiological activities all contribute to sucrose inversions in degraded canes. As per Eggleston [32], the first 14 h of cane deterioration after harvest, are responsible for 93% loss due to microbe invasion, with enzymatic activities accounting for 5.7%, and acid degradation accounting for 1.3%. The weather has a role in such losses as well. Secondary losses can be further sub-divided into the following five types:

i Biological or microbiological losses are caused by insects, fungicides, bacteria, animals, and other factors. The post-harvest pathogens, in particular, do not induce degeneration in healthy canes; rather, they attack when there is damage, such as cuts, cracks, or bruising on the sugarcane stalk. This, in turn, raises the risk of microbe-related losses.
ii Chemical or biochemical losses are those that are caused by primary losses and produce indirect deterioration. Mechanical and physical elements are frequently responsible for these losses.
iii Mechanical losses are a result of mishandling of harvested canes, particularly during transportation.
iv Physical losses occur when adverse environmental conditions are detected for sugarcane. High and low temperatures, low relative humidity, wind, and rainfall are just a few examples. Secondary losses are more common in damp, warm conditions due to the presence of bacteria that release sucrose inverting enzymes [78]. The higher temperature causes more sucrose inversion than when temperatures are low [112]. This is one of the reasons for higher losses in subtropical areas of India ranging from 9.03 to 24.47% [41, 61] than tropical ones ranging from 7.10 to 16.59% [5].
v Physiological losses occur when the natural progression of growth is disrupted, whether in anatomy, physiology, or morphology, as a result of alterations in respiration and transpiration.

## 4 Primary losses

### 4.1 Transportation losses

Man, camel carts, bullock carts, vehicles, trains, trolleys, and other modes of transportation are used to transport sugarcane. Due to a faulty field transport system, the harvested cane is stored in many countries for 3–5 days in the field and 1–3 days in factory storage under unfavourable conditions [47]; [121]. According to Solomon et al. [126], harvested canes brought *via* animal-drawn carts are deemed fresh and of higher quality than those sent *via* cane agencies from various cane centres, regardless of the mode of conveyance utilized. In India, a bullock cart transports roughly 15 to 20 quintals, while a tractor trolley moves about 70 to 80 quintals and a truck transports 170–180 quintals of sugarcane in a single journey [10]. It is a critical factor that influences the quality of harvested sugarcane, as time is the most important factor. However, there are other factors that influence transportation losses, such as the severity of damage in harvested canes (due to mechanical harvesting or loading and unloading), and the size and shape of transport containers.

Grab loaders, push-pile rakes, slings, chains, and other machine equipment elements may also contribute to loss in this category because they cause injury to harvested sugarcane during handling. High temperatures and muddy soils increase the number of injuries and losses (Solomon, 2009). When fresh canes are transported in small containers, the deterioration of harvested canes is less. The long staling periods spoil the quality of canes. Bacteria, particularly *Leuconostoc* are most likely to invade canes picked during the summer months or covered in mud.

In nations like Louisiana, where sugarcane is burnt prior to harvesting, the delay in harvesting time of burnt canes and subsequent storage of these canes creates a congenial environment for the microbe to grow, further increasing transportation losses [118]. Saccharose degradation is harmed by burnt canes, resulting in poorer sugar recovery. Furthermore, regardless of whether the burnt cane is whole stalk or billets, processing of burnt canes within 15 days is required to prevent saccharose fermentation to ethanol [109]. According to Lionnet [79] burnt canes promote wax removal, cane cracks, and juice leakage.

### 4.2 Varietal losses

Sugarcane bio-deterioration after harvest is a varietal trait [21, 42, 44]. Sugarcane types exhibit varying effects on post-harvest sucrose losses. Hall [43] was the first to describe the significance of varieties in post-harvest deterioration, and Egan [31] demonstrated varietal influence on these losses. Different genotypes react differently to post-harvest degradation due to genetic diversity [62, 63]. Varieties are a major component that directly influences sugar recovery, and their impact is further influenced by factors such as environmental conditions and management approaches [75].

The loss of sucrose content in sugarcane is the most significant loss in post-harvest degradation. Fibrous cultivars (such as CoSe 92,423, CoS 97,261, CoS 8432, etc.) are demonstrated to a larger reduction in sucrose level after harvest than those with lower fibre content (such as CoS 95,255, CoS 96,268, CoS 8436, etc.), according to Siddhant et al. [113]. The sucrose reduction is 5.3 percent in CoS 527, 12.54 percent in Co 1148, and 4.62 percent in BO 91 after 24 h of harvest that further increase to 31.17 percent, 55.01 percent, and 30.56 percent, respectively, in March month when temperatures began to rise (after 120 h of cane harvest) [111].

In CoJ 83 (early variety), CoJ 88 (mid-variety), and S70/00 (late variety) a gradual decrease in cane weight, juice extraction%, sucrose%, purity%, and pH has been observed with a concurrent increase in total soluble solids%, titrable acidity index, dextran, reducing sugars, acid invertase, and neutral invertase activity during 12 days of storage [9].

The rate of moisture loss, which is regulated by the different levels of protection afforded against evaporation by genotype-specific stalk features, affects post-harvest degradation [74, 75]. Hard rind and thin cane genotypes are projected to be more resistant to post-harvest degradation, and *vice-versa* [64]. As for resistance to post-harvest inversion, a lot of variances across the cultivars exist [134]. CoC 671 is resistant and Co 62175 is sensitive to post-harvest inversion amongst the six kinds studied [14]. Thangavelu and Rao (1998) also demonstrate CoC 671 resistance to post-harvest inversion. According to studies, not only does the pace of inversion fluctuate from variety to variety, but so does the rate of microbial infection, particularly *Leuconostoc* [17, 24, 71, 124, 137, 139].

## 5 Secondary losses

### 5.1 Microbiological losses

**Fig. 2:**
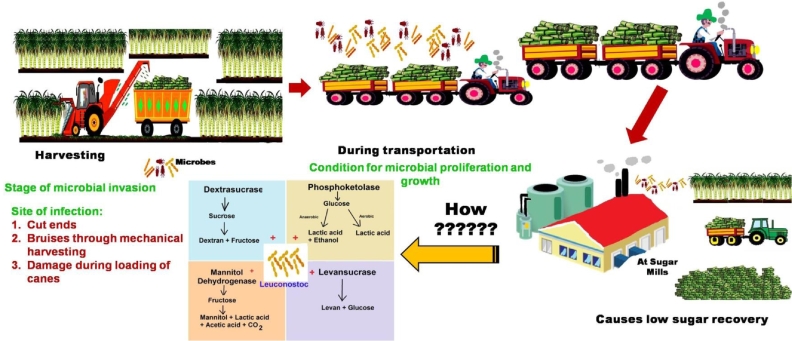
Action of *Leuconostoc* bacteria invasion on sugarcane. The first stage of this bacterium is during harvesting where it infests inside the harvested cane. The second stage of growth and proliferation occurs during transportation to mills and storage at cane centres/mill yards. This, in turn, causes a reduction in sugar recovery at sugar mills by causing difficulties in processing due to the production of various metabolites as this bacterium stimulates the enzymatic activity of four enzymes, *viz*., dextrasucrase, phosphoketolase, levansucrase, and mannitol dehydrogenase.

Microbes play a role in post-harvest sucrose losses as well. They cause changes in host characteristics on invading the harvested canes ([68];  [136]). On entering the harvested stalk through wounds or cut ends, the microbial proliferation thrives in the mature internodes because of their high sugar concentration [129]. Microbial development is also encouraged in leaf sheaths and growing fissures. Bio-deterioration is the term for the process of losses caused by bacteria. Yeast, *Xanthomonas, Aerobacter*, moulds, *Aeromonas, Pseudomonas, Bacillus*, lactic acid bacteria, and other microbes can be found on the outside of sugarcane stalks and are also responsible for the degradation of juice quality during storage [94, 133]. These microorganisms rapidly multiply several weeks after the cane is harvested. Freshly harvested canes harbour a population of heterotrophic bacteria ranging from 1.5 × 10^5^ to 7 × 10^6^ cfu *g* ^−^ ^1^, while stale canes support a population ranging from 9 × 10^5^ to 7 × 10^6^ cfu *g* ^−^ ^1^ tissue [121]. Furthermore, *Streptomyces* bacteria are prevalent in stale cane juice, which causes the sucrose concentration to deteriorate [107]. amongst various bacteria (Yeast, *Xanthomonas, Aerobacter*, moulds, etc.) linked to these losses, *Leuconostoc* sp. is the most common (Solomon, 2009). According to Saxena et al. [108], each gram of acid produced in the canes is either owing to L. *mesenteroides* (2.77 gm of sucrose deterioration) or *E. coli* (11.09 gm of sucrose deterioration). *Leuconostoc* bacteria are soil-borne lactic acid bacteria that infest harvested canes through fissures [90] or cut ends [95]. The invasion of *Leuconostoc* bacterium in harvested sugarcanes has been described as a major sucrose deteriorator in Louisiana, where humid weather conditions prevail. In addition to sucrose as a substrate, this bacterium requires a combination of ambient warmth (> 25 °C) and heavy rains for good and prolific growth and invasion [94]. There have been reports of three species of *Leuconostoc* producing bio-deterioration in harvested sugarcane. These are L. *mesenteroides,* L. *dextranium* (Solomon, 2009; [94, 115]), and L. *lactis* [121], [94, 110, 115]. According to Singh et al. [115], the growth of this bacteria in stale cane juice (cane that has been left out for 20 h) is 370 cfu/100 microlitre, almost double that of fresh cane juice. After the cane is harvested, the infection intensity of this bacterium varies. After 90 min of harvesting, for example, the infection is severe up to 15 cm from the area where the cane is harvested/cut [8]. These bacteria break down the sucrose in the stalk (Fig. 2) and create metabolites such as mannitol, dextran, organic acids, and other compounds [[32], [33], [34], 39].

Sugarcane products are reported to be home to over 400 species of bacteria and fungi (Stevenson and Rands, 1998). After the milling, four microbes namely *Penicillium, Lactobacillus, Leuconostoc*, and yeast are found in stored canes ([47];  [90, 94]). These acid-producing microbes induce degradation and lowering sugar content with reduced juice purity, and pH, particularly in conditions where oxygen is scarce such as mud-coated canes and cane stacked in big piles with little/no ventilation ([121], [95]. Furthermore, enzymatic secretion from these sucrose degrading microbes causes the conversion of reducing sugars to organic acids and mannitol.

### 5.2 Biochemical losses

Invertase activity, dextran synthesis, reducing sugars, and the acidic quality of juice are all biochemical processes that contribute to post-harvest sucrose losses.

#### 5.2.1 Invertase enzyme (Acid and neutral invertases)

Sugarcane post-harvest activities are boosted by the invertase enzyme, which is influenced by cane variety, climatic circumstances, storage conditions, burning, and complicated combinations of these factors [78]. Increased invertase activity [87] causes a reduction in recoverable sugars through reducing mill and boiling house capacity, as well as an increase in sugar loss in molasses, as time passes after harvest due to activation of indigenous invertase enzyme [121] . Acid and neutral invertases are of two types that contribute to post-harvest sucrose losses in sugarcane. Both enzymes are found in cane [103, 104] and their activities get rapid after 72 h of harvest [125].

Acid invertase activity is considerably higher in non-mature internodes [106]. According to Saxena et al. [108], when canes are stored, acid invertase activity increases from 24 to 96 h under ambient conditions, compared to when they are harvested. This rise in enzyme activity ranges between 1.5 and 2.0 folds after 96 h of the harvest [108], but after 12 days of storage, it increases by 7 folds, whereas neutral invertase increases by 4 folds [7]. Chiranjivi Rao [16] and Solomon et al. [125] have revealed a significant drop in sucrose recovery within 72 h of cane harvesting because of an increase in invertase activity. Increased acid invertase activity after harvest could be related to stalk cell invertase enzyme mobilisation, invertase enzyme activation at the cut ends, or activity of enzymes associated with sucrose content activated owing to pH changes [33]. Acid invertase and neutral invertase levels decrease from November to March, according to Bhatia et al. [9].

Siswoyo et al. [116] have discovered the negative role of neutral invertase deteriorating sucrose content particularly in the harvested canes (from the time of harvest till 3 days after harvest). The activity is higher than that of acid invertases, indicating that early sucrose hydrolysis results from neutral invertase activity. Furthermore, Alexander [2] has demonstrated that the upper half of the cane stalk deteriorates faster than the rest of the stalk because it has a larger concentration of inversion-causing enzymes. When compared to newly cut canes, Singh et al. [114] have found a 1.38- and 4.75-fold increase in invertase activity after 48 and 240 h of standing post-harvest canes, respectively. When cane milling cannot be completed within a day of cutting, alternative methods for preserving sucrose content must be used. Suppression of invertase activity has been examined as a possible method for stabilising sucrose concentration at cane maturity and after harvest [121]

Furthermore, a rise in the activity of amylase, acid phosphatase, carboxyl-l-methyl cellulose, and fructose 1–6-diphosphatases, in addition to invertase, leads to a rise in total phosphate and a reduction in starch and sucrose in stale cane [113]. Enzymatic hydrolysis of starch during staling also results in the synthesis of dextran and reducing sugars, causing a rise in juice specific gravity (brix) and a diminution in sucrose recovery [41]. These changes result in an increase in mud volume, a drop in juice settling rate, an increase in molasses output, and a decline in sucrose crystallisation making sugar processing procedures uneconomical [121].

#### 5.2.2 Dextran content

Dextran (a polysaccharide) is a major contributor to sugarcane post-harvest sucrose losses. Huang et al. [51] have demonstrated that increasing the rate of sucrose conversion (13.25 percent – 90.77 percent) when fermentation duration increased from 6 to 84 h results in an increase in dextran concentration from 3.58 to 66.07 mg/ml in mixed juice, resulting in low sugar recovery. Furthermore, the drop in sugar quality and output caused by dextran synthesis is leading sugarcane enterprises to lose money [80].

A soil-borne bacterium known as *Leuconostoc* produces dextran by decomposing sucrose from cane stalks due to the enzymatic activity of dextransucrase. This enzyme breaks the connection between glucose and fructose, resulting in dextran and fructose. This enzyme not only destroys sucrose, but also produces a pH drop [22]. In the sugar mill process, a bacterial complex containing yeast, acetic acid bacteria, and lactic acid bacteria (LAB), including *Leuconostoc* spp., is accountable for the synthesis of dextran from sucrose degradation [23, 48].

Moreover, there are also other bacteria (*Bacteroides, Streptococcus, Pseudomonas* and *Thermoanaerobacter* [38, 66, 85, 148]), fungi (*Paecilomyces lilacinus* and species of the genus *Penicillium, Chaetomium* and *Aspergillus* [36, 37, 85, 143] and few yeasts which cause activation of such enzymes due to which sucrose gets degraded or deteriorated. Furthermore, various issues arise during sugar processing as a result of dextran generation, such as filtration, clarifying, crystal shape alteration, crystallization, viscosity reduction, and so on [22, 59]. In addition, it causes a rise in syrup viscosity [66], degradation in sugar quality [6, 96], and irregular, elongated, opaque, and caramel tone sugar crystals [60].

During the milling process to reduce sucrose losses, dextranases enzymes are used [26, [53], [54], [55]] and to solve problems associated with dextran production, such as grain elongation, boiling period reduction, and smoothness [28, 82]; [60] . The thermostable dextranase enzyme had maximal activity at 80–85 °C temperature, according to Wynter et al. [146].

#### 5.2.3 Acidic nature of juice

Processes in pH and juice acidity after cane harvest indicate that biochemical changes are taking place in stale canes [83]. In general, the pH of harvested cane ranges from 5.2 to 5.3. It has been noted that the pH of juice declines slowly regardless of varietal differences, although the titrable acidity index reveals a reverse pattern in harvested canes [92, 95, 141]. Mathur [88] has recognized that one of the causes of cane deterioration (together with gums and glucose production) is juice acidity. Furthermore, Egan [29] has demonstrated the reason for lowering pH (production of acetic and lactic acids) as an increase in storage duration. Gupta and Nigam [41] have also revealed that drop in juice pH ranges from 5.2 to 5.5 in freshly harvested canes to 4.5 or less in stale canes. A pH of less than 5 indicates cane staling and degradation. When harvested canes are held for 17–27 h, there is a slight loss in pH of cane juice, but an unfavourable decrease in pH is recorded in harvested chopped canes [31]. Furthermore, Solomon et al. [125] have demonstrated 4.2 percent drop in pH on leaving canes in the field for 15 days after harvest. The pH of the juice drops dramatically after 5 days of storage [144].

#### 5.2.4 Reducing sugars

According to Uppal et al. [138], the presence of reducing sugars in juice is a critical factor in the deterioration of stale cane. Several studies have shown an increase in reducing sugars as a result of cane degradation [5, 125]; [101]. A rapid spike in reducing sugars has been documented as a result of the delay in crushing canes after harvesting [29, 41, 49]. The increase in reducing sugars is due to invertase enzyme activity [121] [97]. Parthasarthy [98] has demonstrated that in the months of October (when the weather is cool), a slight increase in reducing sugars occurs in stale canes, whereas in the summer season (when the weather is too hot), reducing sugars rise steeply (three times as much as in normal weather in 72 h stale cane) in the juice of stale canes. Magduma and Kadam [84] have found that the accumulation of reducing sugars is sluggish during the first two days of storage, but accelerates as storage time increases.

Reducing sugars increase from 1.32 to 3.21 percent in stale canes (after six days) [64]. Furthermore, Solomon et al. [125] have found that the accumulation of reducing sugars is not significant up to 72 h after harvest, beyond which a rapid increase in reducing sugars occurs. The level of reducing sugars increases from 0.1 to 3.5 percent after 12 days in stale canes [7]. After 108 h of staling, the high sugar variety Co 9401 also increases reducing sugars [11]. This increase in reducing sugars ranges from 0.32 to 0.90, indicating that it is tolerant to sucrose inversion loss. After 108 h of staling, both CoM 0265 and CoM 0503 have increased reducing sugars at a rate of 20.83 and 25 folds, respectively [12]. After increasing harvest time, CYZ 02–1826 (0.93 percent) and CYZ 02–588 (0.61 percent) indicate a progressive increase in reducing sugars. Furthermore, the Madhurima cane clones 2000 A 105, 2000 A 213, Co 6907, and 2000 A 56 show higher reducing sugars, indicating that the quality of the canes has deteriorated after harvest [97].

The rate of respiration rapidly increases in harvested stale canes that causes an increase in reducing sugars and degradation in sucrose content. The ambient temperature of stacked harvested canes also plays an important part in sucrose deterioration. During winter the temperature in piles of harvested stale canes is revealed to be 0.5–1.0 °C higher while during summers the temperature in piles is 0.5–2.0 °C higher than ambient temperature. The reason is attributed to the production of CO_2_ during respiration that raises the temperature and exacerbates the deterioration.

### 5.3 Mechanical loss (Mechanical harvesting)

Due to degradation in canes, a range of 1–3 percent of the initial sucrose content has been found to be lost. This is also dependent on the harvesting method used . In several places, mechanical harvesting of canes after chopping of burnt canes has been shown to create dextran in juice as well as poor juice quality, resulting in low sugar recovery [30, 31, 56, [70], [71], [72]].

Post-harvest losses in sugarcane due to the usage of a chopper harvester have been reported to be 6–11 percent of average commercial cane sugars in Queensland, Australia. Furthermore, it is high even when compared to previously saved ones [30]. Moreover, Larrahondo [70] has recorded that mechanical harvesting reduces purity and decreases the amount of sucrose and phosphate in canes where garbage has not been removed. Solomon et al. [130] have also found that mechanically harvested cane billets have a 1.0 unit lower commercial cane sugar content than conventionally harvested canes.

### 5.4 Physical losses

Weather has a significant impact on sugarcane sucrose losses after harvest [123, 126, 137, 139]. Temperature extremes, heavy and light rainfall, high and low relative humidity, and so on are examples of weather anomalies. High temperatures (over 40 °C) and low humidity have negative effects on juice quality. According to Solomon [121], the loss of commercial cane sugars varies by season in India. The weather-wise losses are 0.35 units in the winter, 1.0 units in the spring, and 1.32 units in the summer. The warm and dry conditions, thus, incur a heavy loss. The effect of ambient temperature on sucrose losses in sugarcane harvest is well documented. High temperatures during the night enhance the formation of dextran in stored canes [121].

In sub-tropical India, a minimum temperature range of 14–7 °C favours cane juice quality, but when the temperature drops further, sugar concentration drops rapidly. Furthermore, canes harvested in desiccating winds and high temperatures in the months of May and June suffer the most. In general, sugarcane does not dry completely even if the terminal point of growth has died. Nevertheless, the rate of degradation of sugarcane rapidly increases with high relative humidity or a combination of heavy warm weather and frost [149] . During storage, the colour of sugar changes as the temperature and humidity increases [46]. Frost (very low temperatures) is a serious problem in sugarcane processing for sugar companies in several sugar-producing regions, such as Louisiana, Florida, Argentina, and others, where freezing temperatures are common[121] Sugarcane subjected to freezing temperatures suffers severe damage to the stalk, resulting in rapid degeneration. Irvine and Legendre [57] have identified two mechanisms that deteriorate canes during an exposure to cold temperatures. The first is tissue susceptibility in cane stalks and the second is the sensitivity of specific bacteria invading canes. Sugarcane damaged by freezing temperatures shows inferior juice quality, reduced purity, increased acidity, and the development of polysaccharides [33, 77]. According to studies, when ambient temperatures drop below −5.5 °C, the above sugarcane parts die, resulting in enhanced sucrose deterioration. As a result, poor juice quality and low sugar output occur [33, 35, 76]. HoCP 96–540, L 01–283, L 01–299, and HoCP 04–838 are good post-freeze degrading types, while L 99–226, L 03–371, Ho 07–613, L 11–183 are the poor ones.

Rainfall condition indirectly causes sucrose deterioration in sugarcane because muddy conditions favour the production of dextran and the invasion of microbes (particularly *Leuconostoc*, which benefits from the air blockage created by mud covering over harvested canes related to sucrose deterioration). Rainfall during the ripening period results in higher quality and inverted uncrystalllizable sugars in the cane. Rainfall during the harvesting period is harmful to sugarcane and needs to be carefully guarded. The amount of sugar present in cane has been found to decrease from 18.36 percent to 14.15–15.0 percent following significant rainfall [52].

As a result of the challenge of climate change, the diverse abiotic and biotic stresses have become prevalent and diminish productivity and quality of crops with sugarcane being no exception. In sugarcane exposed to abiotic or biotic stresses , the degradation of sucrose begins much earlier. As the time following harvest passes, the rate of sucrose degradation accelerates. The greater the cut to crush delay of such canes, the greater the sucrose losses [92]. The explanation for this is that stress-affected canes have already undergone chemical and physiological changes, resulting in faster sucrose breakdown than normal-grown canes. When water logging occurs in Mauritius due to the humid climate, canes have a higher fructose content. However, sugarcane clones I 133–00 and I 149–00 produce the best results in terms of cane and sugar output, juice quality, and use for breeding under waterlogging conditions [58]. The juice derived from sugarcane flooded with 120 cm deep water from July to September contains lower levels of sucrose [81, 102] and reducing sugars, as well as higher levels of gums [4]. Even non-protein nitrogen (90 percent) is present [67]. In a similar investigation on sugarcane grown in waterlogged conditions, high levels of glucose, total nitrogen, non-protein nitrogen ash, and low levels of P_2_O_5_ have been recorded [4]. When waterlogging is stopped for a month, the sucrose content of the canes increases, and the juice quality improves significantly in the upper area (top) compared to the lower section (bottom). After flooding having been stopped, Co 527 decays sooner than Co 419 and Co 449 [3). Furthermore, sugarcane genotypes Isd-20, I-93/93, and I-8/95 exhibit no significant difference in sugar recovery yield in normal and waterlogged conditions, although juice quality gets slightly better in water logged situations than in normal conditions [45]. Under waterlogging conditions, activities of carbohydrate metabolism enzymes and acidic and neutral invertase enzymes increase significantly, whereas the enzymatic activity of amylase decreases only slightly [81], contributing to low sugar recovery and poor juice quality. As long as water remains standing in the field, these circumstances are accelerated with sugarcane maturity, but as soon as the water is removed, rapid deterioration with excessive concentrations of non-sugars occurs. During the post-waterlogging phase, juice quality deteriorates quickly, but reports suggest that juice quality can be maintained for long periods of flooding as long as the canes remain submerged. On a general basis, flood-survived canes have shown a significant increase in Brix; however, such canes have low sucrose content and high glucose content, resulting in poor juice quality, which hinders sugar recovery. Misra et al. [93] have found that post-harvest deterioration in sugarcane cultivated under waterlogging conditions is faster than those produced under normal conditions and that the juice quality of waterlogging canes is lower than that of normal canes. Furthermore, in a four-year study, the maximal peak of sucrose percent in flooding conditions attains at the 11^th^ month in comparison to the 12^th^ month of cane development in normal conditions, resulting in higher reducing sugars percent in all clones investigated. However, high sugar content cannot be attained because to the least resistance to sucrose inversion 93 WL 1297, 98 WL 1357, 88 WL 2137, 92 WL 1029, NCO 310, and 57 NG 136, which makes it a limiting factor for producing high sugariness under waterlogging conditions [40].

In addition to waterlogging, drought imposes a significant impact on canes suffering from water scarcity. Canes exposed to such a condition show an increase in post-harvest sucrose degradation [91, 92].

### 5.5 Physiological losses

Sugar buildup in sugarcane stalks is one technique to achieve a balance between sucrose synthesis and use. As soon as fully grown and mature sugarcane is harvested, it begins to lose its sucrose content, and the deterioration process accelerates after a few days [92, 95]. This increase in deterioration is due to factors such as temperature fluctuations from day to day, cane burning prior to harvest (though this practice is now less common), injuries from transportation and loading, and an increase in microbe infestation due to a favourable environment for their growth [121] . All of these factors contribute to low sugar recovery, but they also modify sugarcane physiology, exacerbating the problem of low sugar recovery. The following are some of the physiological changes:

#### 5.5.1 Cane weight and moisture loss

Cane weight loss begins once the cane is harvested due to an increased rate of respiration. Several studies conducted at various places and under varying climatic circumstances denote the loss of cane weight attributable to a decrease in the rate of evaporation from the cane stalk surface (Solomon, 2009). Furthermore, there has been a variance in cane weight loss from variety to variety, which is owing to its reliance on factors such as humidity, wind, rain, temperature, and so on. Balsundaram and Bhagyalaxmi [5] have demonstrated that when temperatures are high (during the summer months of April and May), cane weight loss is substantially greater, and when temperatures are low (during the winter months), cane weight loss is negligible. It is commonly known that harvested sugarcane loses very little weight after 24 h of storage. Furthermore, Solomon [126] has observed that in open fields in sub-tropical circumstances, weight loss in the range of 7–10% occurred after 72 h of harvest, while this range increases (7.4–17%) after 96 h, accompanied by a 2.0% loss in sugar recovery at various locations in India.

When canes are stored for 72 h after harvest, chopped canes lose more weight than whole cane stalks [118]. In such conditions, cane weight loss runs from 7 to 10% in open fields [126]. Mehrotra and Sharma [89] have registered a weight loss in harvested canes having a positive correlation with high evaporation rate and high sucrose deterioration. This has a direct influence on cane tonnage (varying from 0.98 percent in cane weight/day to 1.6 percent in sucrose/weight per day), affecting farmer's income [20, 105]. This adds up to the low sugar recovery. A loss of 0.42–1.5 percent in weight each day has been found in sugarcane whole stalk [19]. It is a critical element for Asian countries including India, where sugarcane growers are paid on this basis, causing farmers to lose more money owing to transit delays. According to Solomon et al. [127], the monetary loss to farmers varies between US$ 2.0 and 3.0 per ton of sugarcane delivered to sugar mills.

Weight loss in chopped canes is relatively much higher than whole stalks and this loss becomes apparent within 72 h of harvested cane staling. After 72 h of harvest, Solomon et al. [117] have noted a weight loss of 8.52 and 7.78% in whole cane of two different varieties, CoLk 8102 and BO 91, respectively, under Indian subtropical conditions. Studies report the loss of 3.8–17.89% in cane weight after 72–100 h of harvest in the tropical zone ( [5][14, 17]).

#### 5.5.2 Soil moisture loss

Regardless of the nitrogen dosage provided in the soil, low moisture content (> 5%) in soils where sugarcane is produced causes a continuous drop in juice quality. Optimum soil moisture for sugarcane is 77 percent in field water capacity, never exceeding 60 percent during maturation. The plant absorbs the most moisture during the early stages of crop growth (up to 820 m^3^ per month), and its transpiration coefficient can reach 1200–1500 [121] .

## 6 Management of post-harvest sucrose losses

Sugarcane post-harvest losses can be reduced or decreased if managed properly. In this regard, the following actions must be taken:

### 6.1 General prevention strategies

Knowing the proper harvesting period is very important because it varies depending on maturity/ripening indices. Early-ripening cane should be collected first, followed by mid-late-ripening cane variants. Variation in harvesting conditions on a varietal basis affects these losses as well. L 99–226, L 03–371, Ho 07–613, and L 11–183, for example, demonstrate low cold tolerance and deserve specific treatments while harvesting and crushing. Harvesting of canes should be avoided during hot weather to reduce moisture loss. Harvesting types with a hardy rind or fibre with a high wax content should be favoured in such circumstances [121] .

Sugarcane needs proper handling during the mechanical harvesting, which could otherwise inflict injury to the harvested stalk due to the usage of machinery. Appropriate modes of transportation need to be used. Canes should be loaded in a sanitary and orderly manner to provide proper ventilation in heaps. Harvested canes must be clean of garbage, leaves, and roots prior to crushing. Sugarcane stems that are neat and clean are less likely to deteriorate after the harvest. Cleanliness and hygiene levels should be maintained in cold storage, cane centers, and mill yards. It's crucial to remember the first-come, first-served rule. Mill sanitation is also critical, requiring thorough cleaning and streaming with hot water on a regular basis. Reduced post-harvest sucrose losses have been documented when blades on mechanical harvesters or loaders are appropriately washed/applied with bactericides before the harvesting [18]. Another technique to increase the juice quality of harvested canes left over for several hours is to keep the soil moisture at 10% in loamy soil [121] .

### 6.2 Minimum time duration of cutting to milling

Eagan [27] has laid emphasis on the speed with which the cane is transported to the factory to prevent degradation. When using chopper harvesters, the cane must be crushed as rapidly as feasible. The most practical method of controlling degradation is to cut and grind as soon as possible [135, 142]. It's more important to keep the time between cutting and milling as short as possible.

### 6.3 Sprinkling of water on harvested canes

The best kept cane with minimal loss in sucrose is untopped, covered, and sprayed with water on a daily basis. Water sprinkling alone slows the degeneration, but not as much. After three days, the effect of the covering harvested canes becomes visible. Several studies advocate that before being transported, the gathered cane should be kept untopped, covered, and damp by sprinkling daily. If covering and sprinkling are not possible, the cane should be left untopped and unstrapped until delivery. For a minimum of 7–10 days, sprinkling water over harvested canes held in piles helps in minimizing sucrose inversion [73, 75]. The reduction in cane owing to the use of water sprinkling on a regular basis has been estimated to be around 11% [65, 131]. Furthermore, covering harvested cane piles with cane trash and sprinkling it with water once a day helps reduce post-harvest sucrose losses in sugarcane [98, 100] by causing a constant rate in juice pH of harvested canes [98, 100].

### 6.4 Application of biocidal control

Many studies have documented the use of various biocides and their effectiveness [15]. Ammonium bi-fluoride, quaternary ammonium compounds (QUAT), formaldehyde, thio-carbamates, and halogen compounds are amongst them. Kilbact™ applied at a rate of 20 ppm decreases sugar loss (Solomon, 2009). A continuous mist spraying of organo-sulphur compounds-based formulations on chopped canes in mills improves sugar recovery by 0.5 units as compared to chopped canes without spray and leads to a decrease in invert sugars, acidity, and dextran concentration [119, 124]. According to Coote [18], biocides can help remove 40% of dextran from cane juice. By using disinfectants such as glutaraldehyde and benzalkonium chloride, Singh et al. [114] have obtained a reduction of sucrose loss, enzymatic and microbiological alterations in harvested canes.

### 6.5 Chemical control of postharvest sucrose deterioration

Anti-bacterial and anti-inversion compounds enhance sugar recovery and decrease sucrose losses in harvested sugarcanes. Sprays of benzoic acid (100 ppm) and formaldehyde (100 ppm) are effective against post-harvest sucrose losses [25]. These chemicals retard the post-harvest losses as evident from the changes in brix, sucrose content, cane weight, commercial cane sugar, and other traits. The wettable sulphur has a minor negative impact on the juice quality. Spray of several bactericides, such as ABF, Actin-ID, Kcide 800, Sucroguard, potassium permanganate, and sodium metasilicate, IFOPOL, DNDT, Bactrinol −100, Perla soap solution (1 percent), and others yield promising results in reducing sucrose losses in harvested sugarcanes [118]. Furthermore, allyl isothiocyanate as a key constituent in a solution benefit to reduce sucrose losses after harvest [140].

Another commonly used chemical for minimizing these sucrose losses is sodium metasilicate. Sucrose inversion could be delayed for several days by applying 40–60 mol/ml sodium metasilicate to sugarcane juice samples shortly after milling [1]. The main action of sodium metasilicate seems to be the suppression of endogenous invertases produced by the grinding process from stalk tissue. Fresh juice deteriorated more speedily when fructose or glucose were present, implying that metasilicate had no immediate effect on bacterial invertase other than to deprive the microbes a source of carbon for rapid growth. Complex formation with sucrose molecule is the reason for the prevention of amalgamation of invertase with its substrate when sodium metasilicate is present in low levels in cane juice as chromatographic results indicated. When the rate of this chemical is at a high concentration, complex formation of the chemical at the fructose end takes place. The so-called fructose silicate configuration persists even when sucrose is degraded indicating metabolization of fructose by microbes for their growth and proliferation. The effective retention of fructose by silicate could thus be a bacterial repression mechanism that works in tandem with the invertase inhibitory impact [1].

### 6.6 Chemical formulation usage on harvested canes

Chemicals such as sodium metasilicate/sodium lauryl sulphate as aqueous formulation(s) applied to harvested sugarcanes (whole stalk and billets) reduce sucrose losses due to anti-bacterial properties. Similarly, chemicals such as quaternary ammonium compounds/thiocarbamates exhibit anti-inversion properties [118, 127]. The formulation claims to improve sugar recovery by 0.5 units. Sucrose loss in harvested canes could be minimized up to one week in this manner, regardless of temperature or variety [123]. The formulation of benzalkonium chloride and sodium metasilicate (BKC+SMS) is also effective at high ambient temperatures, with an increase in sugar recovery of 0.3–0.5 units [127]. The combination boosts sugar recovery and commercial cane sugars after 240 h of harvesting, even under drought conditions [92]. Such a formulation is also advantageous for sugar recovery when there is a long delay between harvest and transport to milling [123, 127, 130]. A combination of anti-bacterial and anti-inversion chemicals reduces sucrose losses after harvest. The antibacterial formulation based on organosulphur compounds prevents *Leuconostoc* spp. from generating dextran from sucrose [123]. This would result in a clarifying effect, which would improve the quality of the juice throughout storage.

### 6.7 Use of eco-friendly compounds on harvested canes

Given the health risks of chemicals on sugarcane, using eco-friendly compounds on harvested canes is a useful element in minimizing post-harvest sucrose losses. For example, the use of electrolyzed water (EW) for numerous food processing businesses reduces post-harvest and pre-harvest losses in agricultural commodities and fruits. Treatment with EW of 120 days old harvested canes also manages decline in commercial cane sugars and juice purity (12.23, 82.30, respectively) than untreated canes (9.91, 71.68, respectively) [128]. The author accords the positive results of EW to its extremely effective sterilizing nontoxic biocidal solution against a wide variety of microorganisms in controlling the post-harvest sucrose losses. EW mist spraying on harvested cane storage checks sugar inversion and improves the quality of extracted juice. Huang et al. [51] have applied neutral EW on harvested canes to inhibit L. *mesenteroides* growth (or proliferation) and reduce dextran synthesis and sugar losses.

### 6.8 Other technologies for minimizing post-harvest sucrose losses

In sugar mills, ultrasonic horns are recommended for removing dextran from cane juice produced from stale canes [80]. The technique diminishes sucrose losses after harvest. Vacuum packaging, in combination with preservative treatment, is an effective technique to reduce invertase enzyme activity by minimizing sucrose deterioration and acid accretion at a significant level [118]. Peeled sugarcane has a greater rate of respiration (8.43 times higher than regular sugarcane), according to Mao and Liu [86]. Consequently, keeping peeled canes with a combination of preservative and vacuum packaging is an efficient way of storing for 20 days at 0 °C. Nano vacuum packaging has also a high potential in increasing the shelf life of the peeled and cut canes. During eight days of storage, nano vacuum packaging shows little changes in texture and colour, stable pH value and the total soluble solid content, very small increase in total microbial count, ultimately having an inhibitory effect on polyphenol oxidase (PPO) activity [147]. Tilbury [135] have made dextran free bacteria contaminated cane juice by employing dextranase enzyme, which eliminates about 75% of the dextran in the milled juice.

## 7 Conclusion

Sugarcane is one of India's cash crops, generating revenue for the economy. India is the second-largest producer of sugarcane in the world. Temperature variations in different parts of India where cane is grown have an impact on yield and sugar recovery. This is exacerbated by sugarcane's post-harvest sucrose losses. Sugarcane producers commonly store gathered sugarcane in open fields in stacks before arranging transportation to sugar mills. As a result, a variety of mechanisms that contribute to post-harvest cane deterioration are triggered, resulting in large losses in sucrose content (12 - 50% by crushing stale canes in mills). Primary and secondary losses in harvested canes begin with primary losses related to direct impact on sucrose content in a natural way, while secondary losses are related to the production of secondary products and metabolites such as acids, dextran, and other substances that affect sugar recovery. In the sugar industry, the production of metabolites and secondary products also contributes to the creation of issues during sugar processing and refining. Invasion of bacteria in harvested canes contributes to bio-deterioration in sugarcane. Several bacteria, yeasts, moulds, and other organisms reduce sugar recovery on a large or small scale. Of these, *Leuconostoc* spp*.,* plays a vital role in sugar recovery reduction. It spreads through cut ends (at the time of harvest), grows during transit delays, and reproduces in sugar mills where sugarcane is held in piles before crushing. However, with increased crushing capacity, storage in heaps in sugar mills has been reduced to some extent, but this bacterium has ample time to proliferate during transportation from fields to mills, as well as from fields-cane centers-mills. Cut-to-crush delays must be reduced, and farmers must be made far more aware of losses that occur in their fields or during transit.

There have been a number of common and simple practices, such as early transportation, sprinkling water over-harvested canes and covering them with trash, avoiding mishandling of canes during cutting, loading, and unloading, neat and clean sugarcane (without any dry leaves), loading in the proper way (so that proper ventilation occurs in loaded canes), and so on, that any sugarcane farmer can perform after cane harvest to minimize sucrose losses. These management approaches not only aid in sugar recovery (essential for sugar millers because mill economy is based on sugar recovery), but they also result in less cane weight loss, which farmers are compensated for.
